# Does catching more fish increase the subjective well-being of fishers? Insights from Bangladesh

**DOI:** 10.1007/s13280-021-01698-5

**Published:** 2022-02-15

**Authors:** Sara Miñarro, Samiya Selim, Eric D. Galbraith

**Affiliations:** 1 Institute of Environmental Science and Technology (ICTA), Universitat Autònoma de Barcelona, Building Z-Office 137, 08193 Bellaterra, Barcelona Spain; 2 Centre for Sustainable Development, University of Liberal Arts (CSD-ULAB), House 56, Road 4/a, Dhanmondi, Dhaka, 1209 Bangladesh; 3 Leibniz Centre for Tropical Marine Research (ZMT), Bremen, Germany; 4 Institució Catalana de Recerca I Estudis Avançats (ICREA), Barcelona, Spain; 5 Department of Earth and Planetary Sciences, McGill University, 3450 University Street, Montreal, QC H3A 0E8 Canada

**Keywords:** Affect, Ecosystem services, Positive psychology, Small-scale fisheries, Social-ecological systems, Subjective well-being

## Abstract

Small-scale fisheries have been associated with the subjective well-being of coastal communities through their links with culture, identity, and social cohesion. But although fish catches are usually considered the primary ecosystem service that benefits fishers, little is known about how subjective well-being is influenced by the fishing activity itself. Here, we applied the experience sampling method in two small-scale fisheries in Bangladesh to assess the effects of fishing on fishers’ occurrence of positive and negative affect, two measures of subjective well-being. We found that fishing activities were not directly associated with increased momentary affect and that the frequency of positive affect actually decreased as the fishing trip progressed. Furthermore, although very low catches were associated with less positive affect, the highest frequency of positive affect was achieved with relatively small catches. Our results imply that the benefits provided by small-scale fisheries to the momentary subjective well-being of fishers are not strongly related to the actual catching of fish.

**Supplementary Information:**

The online version contains supplementary material available at 10.1007/s13280-021-01698-5.

## Introduction

Small-scale fisheries are considered a pillar of socio-economic well-being for millions of people in coastal communities (The World Bank 2012; Teh and Pauly 2018). These diverse fisheries generate most of the catch for direct human consumption in low-income countries, providing essential food and income. In addition, small-scale fisheries are deeply associated with people's identity, social norms and lifestyle, all of which create a strong attachment to fishing (Pollnac and Poggie 2008; Cinner 2014). Fishing has been associated with subjective well-being—feelings of happiness and satisfaction—because fishing activities are linked with communities’ culture, identity and social cohesion through multiple dimensions (Weeratunge et al. 2014; Allison et al. 2020). But the increasing commercialization of small-scale fisheries, leading to profit-driven overfishing and the subsequent degradation of coastal fish stocks and natural marine environments, threatens the societal benefits of this ancient human-nature interaction (Lapointe et al. 2021). Furthermore, it has been suggested that the profit- and yield-focused assumptions underpinning fisheries management have produced a myopic paradigm where the most important benefits from fisheries to humanity are overlooked (Allison et al. 2012).

Fishing can influence well-being both at the individual and societal level. Individual influences can arise from engaging in physical activity in nature, living adventures and overcoming challenge (Thompson Coon et al. 2011; Holland et al. 2020). Fishing can provide a fulfilling social status, particularly in small-scale societies, associated with the opportunities to display strength and bravery, cooperate with others, and provide for one’s family and community (Naranjo-Madrigal and van Putten 2019). Being a “good fisher” is associated with features that develop social cohesion, such as food sharing, frequent interaction and communication, and reciprocity at sea (Gustavsson et al. 2017; Holland et al. 2020). Fishing is commonly understood as a “way of life”, and the pride and self-actualization obtained from being a fisher has been found to be the main explanation for fishers’ reluctance to stop fishing, even when stock depletion causes it to be unprofitable (Pollnac et al. 2012). Fishers seem to have adapted to the risks of the fishing profession -one of the most dangerous occupations in the world (Kaplan and Kite-Powell 2000; Håvold 2010), and have reported feeling a thrill that fulfills their fundamental need for adventure (Pollnac and Poggie 2008). Furthermore, people in small-scale fishing communities have cited fishing among the top things that bring them most happiness (Miñarro et al. 2021). These results could be assumed to imply that catching more fish would yield more positive emotions (e.g., pride, happiness), and thus greater fishing effort would bring greater well-being to small-scale fishing communities.

However, little empirical research exists on the mediating factors between engaging in fishing activities and increased subjective well-being (Weeratunge et al. 2014; Johnson 2018). Subjective well-being (hereafter SWB) is recognized as an important component of the social well-being approach in fisheries (White 2008; Coulthard et al. 2011; Johnson 2018). This approach envisions three dimensions of well-being including the material, relational and subjective dimensions, and has been used as a framework to investigate the locally relevant well-being benefits derived by fishing communities from coastal ecosystem services (Coulthard et al. 2014; Lau et al. 2019; Lapointe et al. 2021). Recent research has focused on how specific stages of the small-scale fisheries value chain may provide well-being benefits to different stakeholders (Galappaththi et al. 2021). While this has provided important findings on the varied nature of benefits and the mediating effects of specific factors, such as urbanization, discrimination or management approaches, a substantial data gap remains in mechanistically understanding how these benefits from the environment directly relate to the experience of human well-being (Carpenter et al. 2009; Lapointe et al. 2021; Fabinyi and Barclay 2022). One little explored way to do it is by quantifying the effects of a specific activity on fishers’ subjective well-being at the individual level (Soga and Gaston 2020).

SWB entails people’s appraisals of their own lives, including both reflective cognitive judgements, such as life satisfaction, and emotional responses to ongoing life events (i.e., positive versus negative emotions) (Diener et al. 2018). These two dimensions are only moderately correlated and have been shown to provide complementary information on the overall SWB (Diener 2009). Emotions are thought to be short-lived reactions that are tied to specific events or stimuli (Kahneman et al. 1999), and “affect” is the experienced set of emotions felt by a person at a particular moment, which is usually associated with the context in which the person is embedded (Diener 2009). Affect appears to be causally related to success, health and longevity in both men and women (Lyubomirsky et al. 2005; Steptoe and Wardle 2011; Pressman et al. 2019), and positive emotions during sequential exchanges are associated with desirable social features such as increased commitment and group cohesion (Lawler et al. 2008).

The emotions elicited in a person by a given situation can be pleasant and unpleasant, yielding positive affect (PA) and negative affect (NA). PA and NA are independent and affected by different variables and should therefore be measured separately (Bradburn 1969; Diener 2009). Momentary affect can be measured through the Experience Sampling Method (ESM) (Csikszentmihalyi and Larson 2014). By prompting participants to answer questions about their current emotions and activities, this measure avoids recall bias and undergoes relatively little filtering against learned standards and expectations (Shiffman et al. 2008). Because ESM yields repeated measurements of a person’s activities, emotions, thoughts, and motivations over time, it can help reveal how much of the person’s variation in happiness (or any other state) is related to what the person does, the company they keep, the time of day, etc. (Csikszentmihalyi and Larson 2014). ESM thus provides a direct quantification of how the activities a person does affect their well-being in real time.

Positive affect appears to be strongly influenced by personal characteristics and temperament (e.g., sleep quality, personality traits) and by local features of the current situation, while general circumstances, such as income or education, have little impact on the momentary experience of a regular day (Kahneman et al. 2004). Diurnal cycles of affect and tiredness have also been reported by different studies (Stone et al. 1998; Kahneman et al. 2004). Furthermore, negative affect has been shown to peak in the morning and then decrease throughout the day (Kahneman et al. 2004). Some studies found marked contrasts between the expected enjoyment provided by specific activities, and the actual affect reported when doing the same activities, which highlights the difference between belief-based generic judgements and specific episodic reports (Kahneman et al. 2004; Shiffman et al. 2008; Diener et al. 2009).

Of potential relevance to fishers, correlational studies have found transient positive affect during challenging, highly skilled activities to be associated with a rewarding state called flow (Csikszentmihalyi 1990; Bryce and Haworth 2002). Flow is a state in which people are completely immersed in an activity in which their level of skill matches the challenge at hand, and where “action follows upon action according to an internal logic which seems to need no conscious intervention on our part” (Csikszentmihalyi 2014). In flow, the activity itself is intrinsically rewarding, independently of external incentives, and leads to a sense of control and positive affect. For example, activities such as rock climbing, chess, or writing can lead to flow state, “the optimal experience”. Although individuals differ in their propensity to achieve flow, activities must meet certain conditions for flow to occur: they must contain a clear set of goals, there must be a balance between the perceived challenges and perceived skills, and there must be clear and immediate feedback (Nakamura and Csikszentmihalyi 2014). The reported self-actualization component of fishing, including “adventure” and “challenge” (Pollnac and Poggie 2008; Pollnac et al. 2012), coupled with a clear goal and the high skill required from fishers, could indicate that fishing commonly leads to a flow-like state (Vittersø 1997).

Here we investigate how fishing influences momentary affect by assessing the experienced well-being of fishermen in two small-scale fishing communities in Bangladesh. We specifically ask: (i) how does the momentary affect of fishermen vary among their daily activities?, (ii) is fishing success associated with fishermen’s affect during or directly after a fishing trip, and do the predominant emotions change with fishing success?, and (iii) can we detect the occurrence of flow during challenging and highly skilled fishing activities? Our results test the hypotheses that (i) activities associated with fishing would produce more positive affect than non-fishing activities, (ii) the frequency of positive affective states and emotions is proportional to the fish catch rate, and (iii) activities meeting the conditions for flow will be associated with more positive and less negative affective states. By using well-established psychological tools to test the association between fishing and affect, our work helps expand the understanding on the mechanisms by which fisheries support the well-being of fishing communities.

## Materials and Methods

### Study sites

In Bangladesh, an estimated 86% of marine catch is produced by artisanal fisheries (Bangladesh Bureau of Statistics 2016), highlighting the importance and ubiquity of this sector nationwide. Hilsa (*Tenualosa ilisha*), considered the national fish of Bangladesh, makes up an important part of Bengali cuisine and is the most important target species (Bangladesh Bureau of Statistics 2016). The purpose of fishing is primarily for sale, although fishermen tend to keep a small part of the catch for family consumption, and some of it is destined for export. This study is part of a broader research project for which the lead author spent several weeks living in each of the studied communities, coordinating the research team and interviewing key informants (Miñarro et al. 2021). The information in this section comes from field diaries and own ethnographic data collected in the framework of this work, combined with published literature when available.

**Fig. 1:**
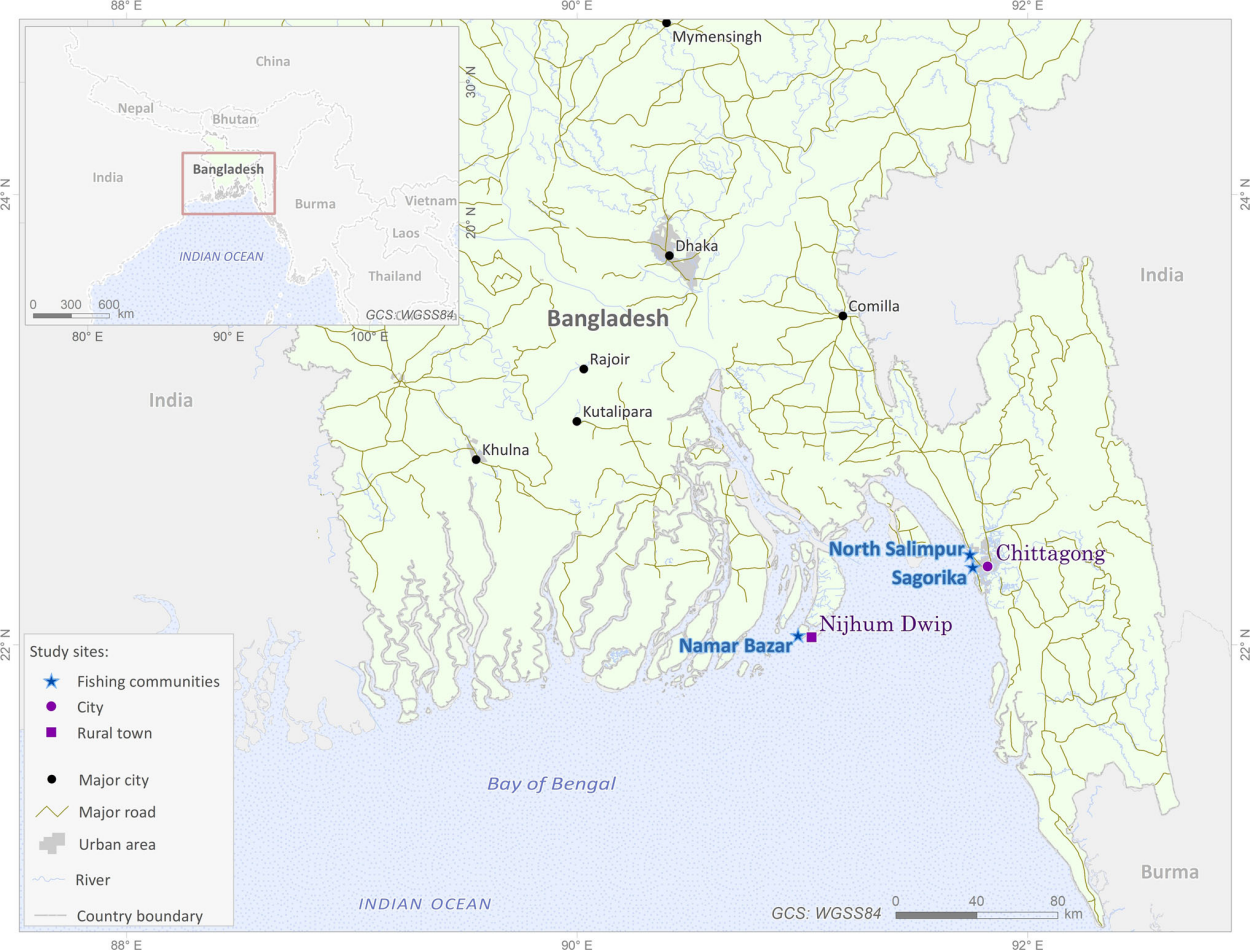
Map of the study sites. Sources: Administrative boundaries (GDAM, https://gadm.org/), Rivers and Cities (MapCruzin.com (OpenStreetMap data courtesy of http://download.geofabrik.de), Urban areas (obtained from Global Land Cover 2000, original data resampled onto a 30 s grid, https://www.diva-gis.org/gdata), and Roads (Digital Chart of the World, https://www.diva-gis.org/gdata)

The data collection took place in two different sites, one rural and one urban, to control for the effect of urbanization in the benefits derived from fishing (Lapointe et al. 2021). The rural site in this study is Nijhum Dwip (Fig. 1), a remote island created by a sand alluvium accumulation and first colonized by fishermen in the early 1950s. We surveyed fishermen from the small landing site of Namar Bazar (Fig. 1). The majority of the population in Nijhum Dwip adhere to Islam, and its inhabitants face precarious living conditions (Siam et al. 2020). The island is highly vulnerable to climate change impacts and is frequently flooded by monsoonal storms and cyclones (Government of the People’s Republic of Bangladesh 2017). The fishing is done exclusively by men around the island using push nets, bag nets, and gillnets. Most fishermen work for a patron, who provides a loan, boat and fishing gear, and the fish market is controlled by patrons. Fishermen in Nijhum Dwip went on fishing trips during 4.2 ± 2.8 days per week, and mostly did one long (5–8 h) fishing trip per day. Occasionally, fishing crews go to distant fishing grounds requiring trips of up to a week. The Nijhum Dwip small-scale fishery is very risky; fishermen are often caught in monsoon storms with no assistance due to the lack of rescue fleet coverage in the area, according to the key informants, and there is no hospital on the island. Furthermore, institutionally sanctioned piracy was anonymously reported to this study’s researchers on a number of occasions, leading to economic losses, personal harm, and losses of fishing equipment. Communities depend on money for certain goods and services, and at least one household member typically engages in some form of paid work, but all households are involved in subsistence activities.

The urban site is within the Chittagong area, the largest port in Bangladesh and the second largest city in the country. We surveyed two communities in the metropolitan area, North Salimpur and Sagorika (Fig. 1). Fishing in these communities was dominated by Hindu fishers, who consider fishing as their identity and tradition. Fishing at both sites is done with gillnets on designated permanent fishing grounds that fishers reach with engine boats. Fishermen fished 5.5 ± 2.4 days per week, with each fishing day including between 3 and 4 fishing trips of about 4 h each. The fish is sold to fishmongers at the landing site and taken to be resold in the Chittagong city markets. Only men are involved in fishing, but some women work as fishmongers and maintaining the nets. While North Salimpur is a small, close-knit permanent Hindu community, nearly half of Sagorika’s workforce is comprised of immigrants from other parts of Bangladesh, mostly Muslims, coming as temporary fishing labor for the 6-month hilsa season.

At both sites, participants were full-time fishermen, typically organized in fishing crews, i.e., the crew working on the same boat during the fishing season. Net repair is a time-consuming daily task of fishing crews in Nijhum Dwip, but it is usually a task done by someone other than fishermen in Chittagong, typically fishermen’s wives or men who are hired for it. A previous study on these sites found that the studied Bangladeshi communities have high subjective well-being relative to the national average (Miñarro et al. 2021). Further, fishermen often related different aspects of the fishing profession (notably high catches, among others) when prompted about what makes them happy (Miñarro et al. 2021).

### Sampling and data collection

Data collection took place between April and July 2018. Participants were informed of the nature and purpose of the study and agreed freely to participate understanding that they could withdraw from the research at any time without consequences, giving their oral consent.

Participants' momentary affect was assessed via the Experience Sampling Method (ESM), which consists in surveying research subjects at random times throughout the day (Hektner et al. 2007). Volunteer fishermen were recruited in Nijhum Dwip (N = 27) and Chittagong (N = 20). A summary of the ESM sample is shown in Table S1; because of the intensive sampling technique and to adhere to anonymity ethical requirements, only the age of respondents was recorded. Participants were all male due to a lack of female involvement in fishing activities, and their mean age was 32.8 ± 12 years old. Participants freely agreed to provide their mobile phone number and answer the phone every time they were called. Fishermen in the sample were called at random times twice a day (morning and afternoon/evening) for one week in July and one week in August 2018 and asked about their current activity and what emotions they were feeling.

To record their affect, participants were offered a list of positive emotions (*Happy*/*Smiling*/*Well*/*Satisfied*), negative emotions (*Angry/Anxious/Worried/Not good at all/Scared*), and physical states (*Hungry/Thirsty/Ill/Sick/Tired*), and they could choose as many as they felt fit their current state or respond freely with emotions not present on the list. The list was developed before going to the field based on common pleasant and unpleasant emotions (Diener 2009) and refined during a pilot study with small-scale fishers in the Solomon Islands (Miñarro et al. 2021). During pilot testing, several respondents alluded to physical states when asked “What are you feeling right now?”, so the option of answering with physical needs was added. However, these are not considered for the affect calculations. Participants were also asked to report what they were doing at the moment (i.e., their current activity, which was open-ended), why they were doing it (*I have to/I want to/I have nothing else to do*), how challenging the activity was (*Very/Quite/A little/Not at all*) and how skilled they considered themselves at their current activity (*Very/Quite/A little/Not at all*). If they were fishing at the time of the call, they were asked to specify how long they had been fishing (open-ended), whether the fishing was going well, whether they had caught anything, and what was the nature of their current catch (species and weight) to evaluate the possible effect of these fishing variables on their affect. The study was given ethical approval by the UAB ethics committee under reference CEEAH 4119.

### Data analysis

Emotional state responses were classified into three “affective states”: (1) positive affect (PA) including happy, well, smiling and satisfied; (2) negative affect (NA) including worried, anxious, angry, not good, scared; and (3) physical needs including hungry, sick, ill, thirsty, and tired. For each call, positive affect, negative affect, and physical needs were coded as present or absent. If a fisherman reported one or more emotions/needs in an affective state, that affective state was recorded as present. Absent indicates no emotions/needs were selected for an affective state. Because of this approach, more than one affective state could be recorded per call. While some ESM studies have asked participants to rate the intensity of a given set of emotions (e.g., (Larson and Csikszentmihalyi 2014), it is also recommended that the contents on the questionnaire are targeted to the research goals and cultural setting (Diener 2009; Nakamura and Csikszentmihalyi 2014). We chose the presence/absence approach to reduce the response burden and ensure participants remained motivated to answer the calls. Furthermore, our pilot tests showed that many respondents were not inclined to give a graded response but rather they themselves only contemplated the presence or absence of emotions.

Activities were inductively categorized into various fishing-related categories, resting, and non-fishing activities (Table 1). This was done through peer coding, where two researchers classified all the responses independently and then compared and discussed their resulting categories until consensus was reached (Bishop and Dzidic 2014). Given the study’s focus on subjective experience, we tried to keep the fishermen’s own classification of activities whenever possible. During this process, it became apparent that “fishing” comprised different phases throughout the fishing trip, which displayed marked differences in affect responses. Fishing preparation refers to when fishermen were getting ready to go fishing. Boat and net repair were treated as separate activities because while net repair is a fundamental part of the fishing operations in Bangladesh, boat repair is uncommon, taking place only when an accident occurs. A category called “fishing-related unspecific” was created for when participants answered only “work”. Because participants were full-time fishers, and the answers tended to be specific when they were doing other non-fishing-related activities, we assumed *work* refers to fishing-related tasks without further specification.

**Table 1:** Activity categories, their frequencies of occurrence among all calls, and representative examples of the textual responses provided during the calls

Activity categories	Observations (%)	Response examples
Fishing	271 (28%)	“Fishing”, “on the boat fishing”
Non-fishing activities	263 (27%)	“Eating”, “shopping”, “talking”, “drinking tea”, “walking”, “playing”
Resting (control activity)	162 (17%)	“Resting”, “sleeping”
Selling fish	71 (7%)	“Selling fish”
On the way to fish	55 (6%)	“Going fishing”, “on the way to fishing ground”
Returning from fishing trip	48 (5%)	“Back from fishing”, “going home after fishing”
Repairing boat	31 (3%)	“Fixing boat engine”, “repairing the boat”
Repairing net	30 (3%)	“Repair net”, “sewing net”
Fishing-related, unspecific	21 (2%)	“Work”
Fishing preparation	17 (2%)	“Preparing to go fishing”, “eating and getting ready for fishing”

A Pearson correlation was used to test the correlation between PA and NA. Generalized linear mixed models (GLMM) with binomial family were applied to test if the occurrence of positive and negative affect (the dependent variables in the models) was different depending on respondents’ current activity, time of the day, call number, site, the reason they gave for doing the activity, their perceived skill level, and the activity difficulty (as explanatory variable in individual models). We tested for and found no differences between the two sites in reported positive or negative affect, and thus the location variable was not considered in the final analysis. Participant ID and call number were considered as random effects to account for repeated measures on the same set of participants, and only participants with 6 or more calls were included in the models. Odds ratio tables were calculated for the activities, which had significantly different PA and NA for their categories, using “resting” as control activity. Resting (including sleeping) was chosen as control because it was presumed to be neutral in that, unlike in the other categories, participants do not receive much input from the environment that would influence their affect. Chi square tests were used to evaluate differences between the study sites in the explanatory variables.

To evaluate the role of fishing success on fishermen’s affect, we used GLMMs with repeated measures to evaluate two types of success: subjective evaluation and objective fishing production. First, we address whether fishermen’s own evaluation of the fishing trip at the time of the call influences the incidence of positive and negative affect. Second, we tested the relationship of positive and negative affect with fishing trip production in terms of absolute catch in kg and CPUE (catch per unit of effort) as fishing efficiency in kg per hour. To avoid potential confounding effects arising from variable market values, only fishing trips exclusively reporting hilsa in their catch were considered (*N* = 220), while fishing trips reporting other species in the catch were excluded (*N* = 75). Radar charts were produced to display the occurrence of disaggregated emotions for each of the three categories of subjective evaluation of the fishing trip to further explore the connection between the two variables. Because physical needs did not show enough variation across activities, it was not possible to run GLMMs with their occurrence as a response variable. Thus, they were excluded from the activity analyses, although we use them as descriptive additional information to characterize the occurrence of individual emotions.

To evaluate the presence of conditions for flow and their effect in the occurrence of positive and negative affect, each call was classified according to their combination of perceived challenge and skill following Csikszentmihalyi’s Flow Model scheme [(Csikszentmihalyi 1990), reproduced in Fig. 5B]. Next, the proportions of reported positive and negative affect, and the reason for doing activities, were assessed for each combination of challenge and skill.

All statistical analyses were carried out using R, version 3.5.2, and the “lme4” package (Bates et al. 2014) was used to carry out the GLMM analyses. Average values are reported as mean ± standard deviation and p-values are considered significant when < 0.01 throughout the manuscript.

## Results

### Emotional states and activities

We were able to contact participants 1075 times. Interruptions occurred during 92 of the calls, leaving 983 complete phone interviews. We obtained between 1 and 31 interviews from each participant (mean: 20.7 ± 9.5). Overall, 52.6% of the calls reported positive affect, while 30.5% reported negative affect, and these proportions were virtually identical at both sites. The remaining 16.9% did not report any positive or negative affect, only physical needs. Positive and negative affect were negatively correlated (*R*^2^ = − 0.68), with only 4 of the calls reporting both positive and negative affect. *Worried* was the most frequent negative emotion reported by Bangladeshi fishermen (Fig. S1). *Well* and *happy* were the most commonly reported in the positive emotions’ category. *Hungry* was the most common of the physical needs, except for being *sick* which was frequently reported by participants who were resting. The incidence of responses indicating positive and negative affect was not significantly associated with site, time of the day, or whether fishers felt any sort of physical discomfort, while the study site and whether fishers felt any physical discomfort were significantly correlated only with reports of physical needs (Tables S2–S4).

Table 1 shows the activity categories with examples of textual responses given by participants. The most frequent activity during our calls was *fishing*, followed by *non-fishing activities*. The least common activities (< 5%) were *fishing preparation*, *fishing-related-unspecifi*c, *net repair,* and *boat repair*. Frequency of some activities was different between both sites; *fishing* was more frequent in Chittagong (31.7% versus 24.9% in Nijhum Dwip) while *resting* and *other activities* were more frequent in Nijhum Dwip (resting: 12.9% Chittagong vs 19.9% Nijhum Dwip; other activities: 23.7% Chittagong vs 30% Nijhum Dwip). Net repair, although infrequent at both sites, was far more common in Nijhum Dwip (5.1% vs 0.7% in Chittagong).

### Differences in fishermen’s affect while engaging in fishing-related activities vs. others

**Fig. 2:**
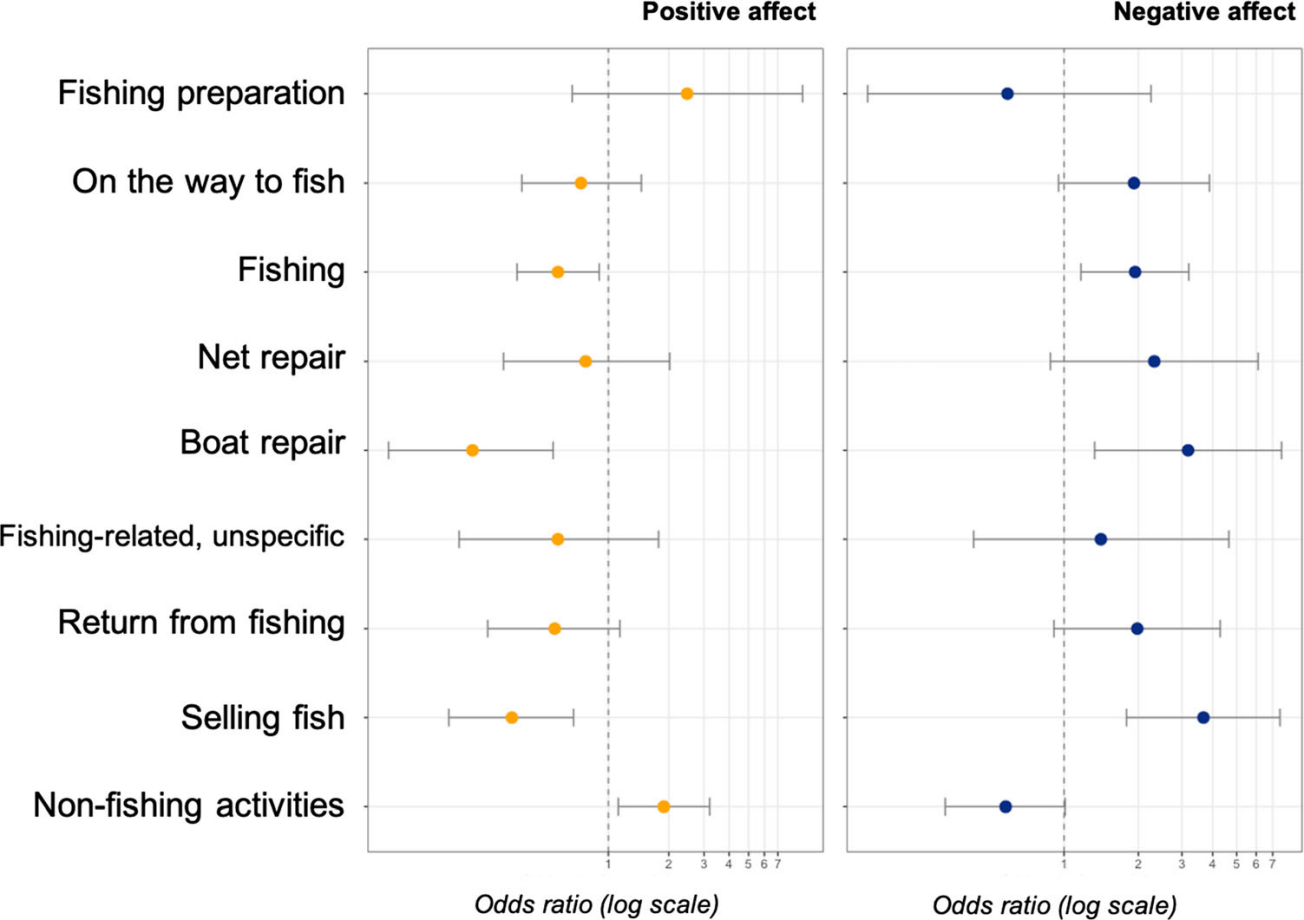
Odds of fishers reporting positive and negative affect depending on the activity they are doing. The reference for comparison is *resting*, which was more positive than most activities (65.5% report positive affect, while 27.4% report negative). Error bars indicate 95% confidence intervals

We calculated the odds of a fisherman reporting positive or negative affect according to the type of activity in which he was engaged as compared to the control activity, *resting* (Fig. 2, Table S5). In general, there was a strong negative correlation between the odds for positive vs. negative affect by activity. *Fishing preparation* was the only fishing-related activity more likely to be associated with positive affect than the control activity, and the difference was not statistically significant. Furthermore, the odds of reporting positive emotions decreased as the fishing trip phases progressed, i.e., *fishing preparation*, *net repair*, *traveling to the fishing ground*, *fishing*, *returning from fishing* and *selling fish* were gradually associated with less frequent positive and more frequent negative affect. Particularly negative affect-producing activities were selling the fishing catch and doing boat repairs.

### Role of fishing success on fisher’s momentary affect

**Fig. 3:**
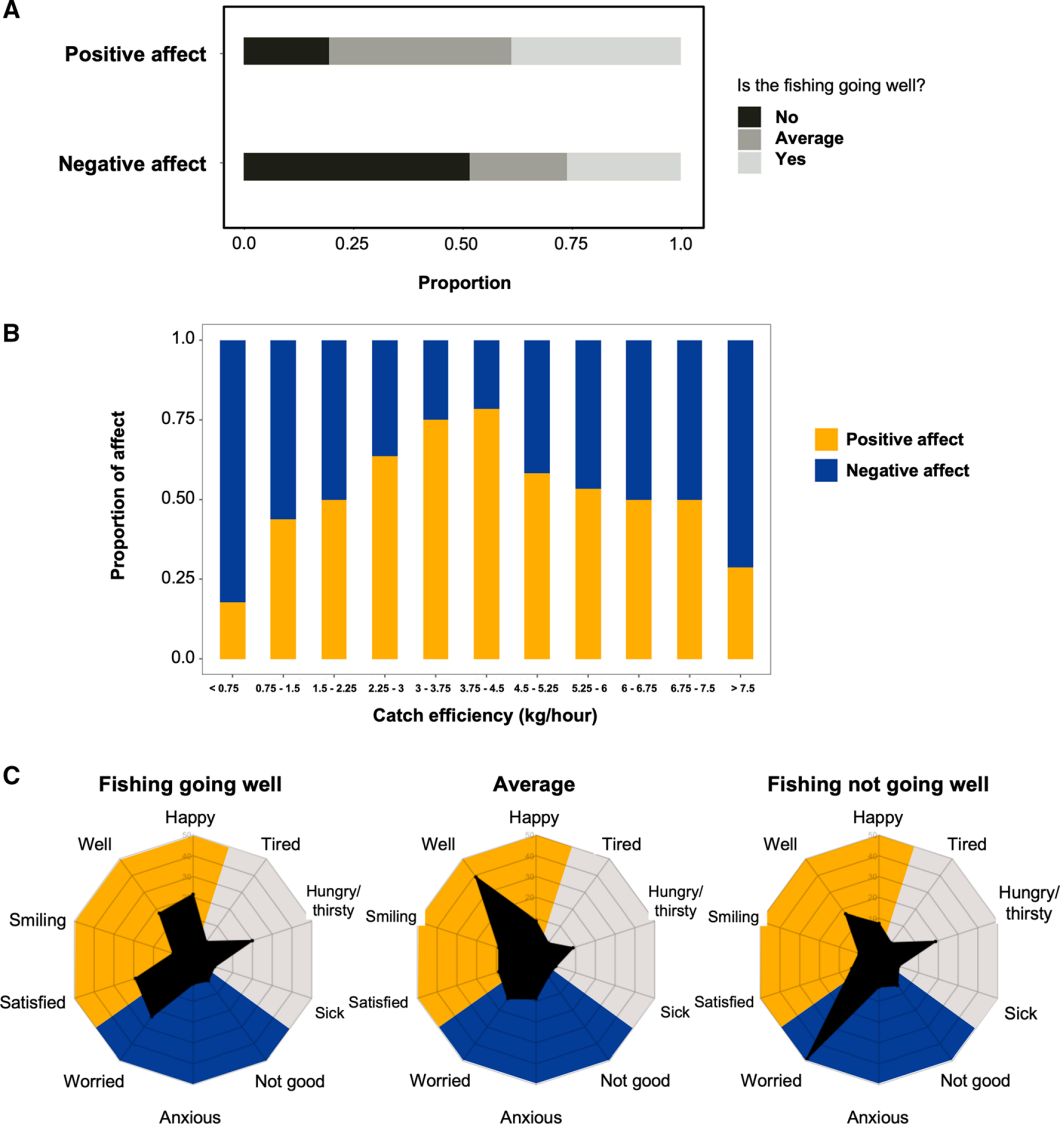
**A** Response distribution of perceived fishing success for calls reporting positive and negative affect. **B** Share of positive and negative affect with catch efficiency for hilsa fishers. **C** Proportions of reported emotions and physical needs reported along with different fishing trip evaluations (yellow: positive emotions, blue: negative emotions, gray: physical needs)

The response of fishermen to the question “Is the fishing going well?” was significantly correlated both with positive and negative affect (Tables S2, S3), suggesting that the subjective assessment of fishing success is linked to affect (Fig. 3A). In contrast, after accounting for repeated measures, we did not find a significant overall difference in objective measures of catch (the absolute catch and catch rate) between fishermen who reported positive vs. negative emotions. Despite the lack of linear correlation, when the proportion of positive vs. negative responses was compared for different catch rates, it revealed a dominance of negative affect at very low rates of catch (< 0.75 kg h^−1^), increasing rapidly to reach maximal values at rates of 3.75 to 4.5 kg h^−1^, and then plateauing or decreasing at very high rates (as high as 12.5 kg h^−1^, Fig. 3B).

Our results also reveal clear associations between emotions and subjective evaluations of fishing trip success (Fig. 3C). Calls which reported that the fishing trip was going well reported dominantly positive emotions (*satisfied*, *well* and *happy* make up 58% of responses), with some reported being *worried* (23.8%). Calls which reported that the fishing was not going well were dominated by the emotion *worried* (48.5%), with a small number of *well* (17.2%) mentions. Mentions of *hungry/thirsty* were similar among calls regardless of whether the respondent reported that fishing was going well or not (19.9% and 18.6%, respectively). Calls reporting that fishing was average were dominantly associated with the positive but also relatively neutral response *well* (39.1%).

### Flow-related aspects: Reasons for doing activities and challenge-skill balance

The most common reason given for doing the current activity was *I have to* (52.7%), followed by *I want to* (36.6%) and *I have nothing else to do* (10.7%). The proportions were different between sites (*χ*^2^(2) = 15.5, *p* = 0.0004), with urban Chittagong having more frequent *I have to* answers (59.4% vs 47.3% in Nijhum Dwip), and rural Nijhum Dwip having higher frequencies of *I want to* (39.6% vs 32.9% in Chittagong) and *I have nothing else to do* (13.1% vs 7.7% in Chittagong). With regard to perceived difficulty of activities, most calls reported the activity participants were doing was *not challenging at all* (43.4%), with a large disparity between the sites (*χ*^2^(3) = 35.1, *p* < 10^–6^). In Nijhum Dwip, 51.2% reported activities being *not challenging at all*, followed by *very challenging* (17.5%). On the other hand, in Chittagong *not challenging at all* was reported in 33% of the calls, followed by *quite challenging* (25.8%) and *very challenging* (24.8%). Most fishers reported being quite or very skilled at their current activities, with little variation between the sites (*χ*^2^(4) = 10.9, *p* = 0.03).

**Fig. 4:**
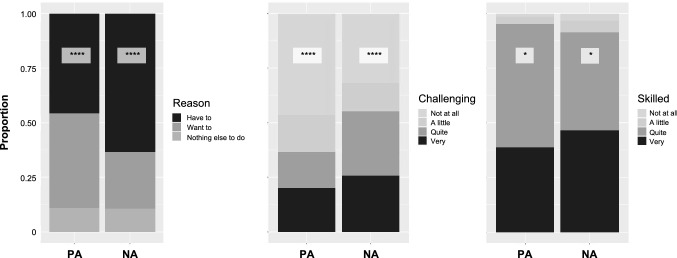
Proportions of responses rating **A** the reason for doing activities, **B** how challenging participants perceived their current activities, and **C** participant’s skillfulness self-assessment for activities that co-occurred with positive and negative affect. The asterisks indicate significance as follows: *p ≤ 0.05, **p ≤ 0.01, ***p ≤ 0.001, ****p ≤ 0.0001

The reason given for doing the current activity, and how challenging participants considered their current activity were both correlated with different affect responses (Fig. 4, Tables S2, S3). The reason *I want to* was more likely to yield positive affect, and was less than half as likely to be associated with negative affect than *I have to* (Fig. 4A). Activities considered *not* or *a little challenging* were more likely associated with less negative and more positive affect than activities considered quite or very challenging (Fig. 4B). Fishers’ own perceived skillfulness at activities was barely significant (Fig. 4C), owing to the little variation in responses. Figures S2 and S3 show the distribution of reasons and the perceived challenge proportions given by each activity category.

**Fig. 5:**
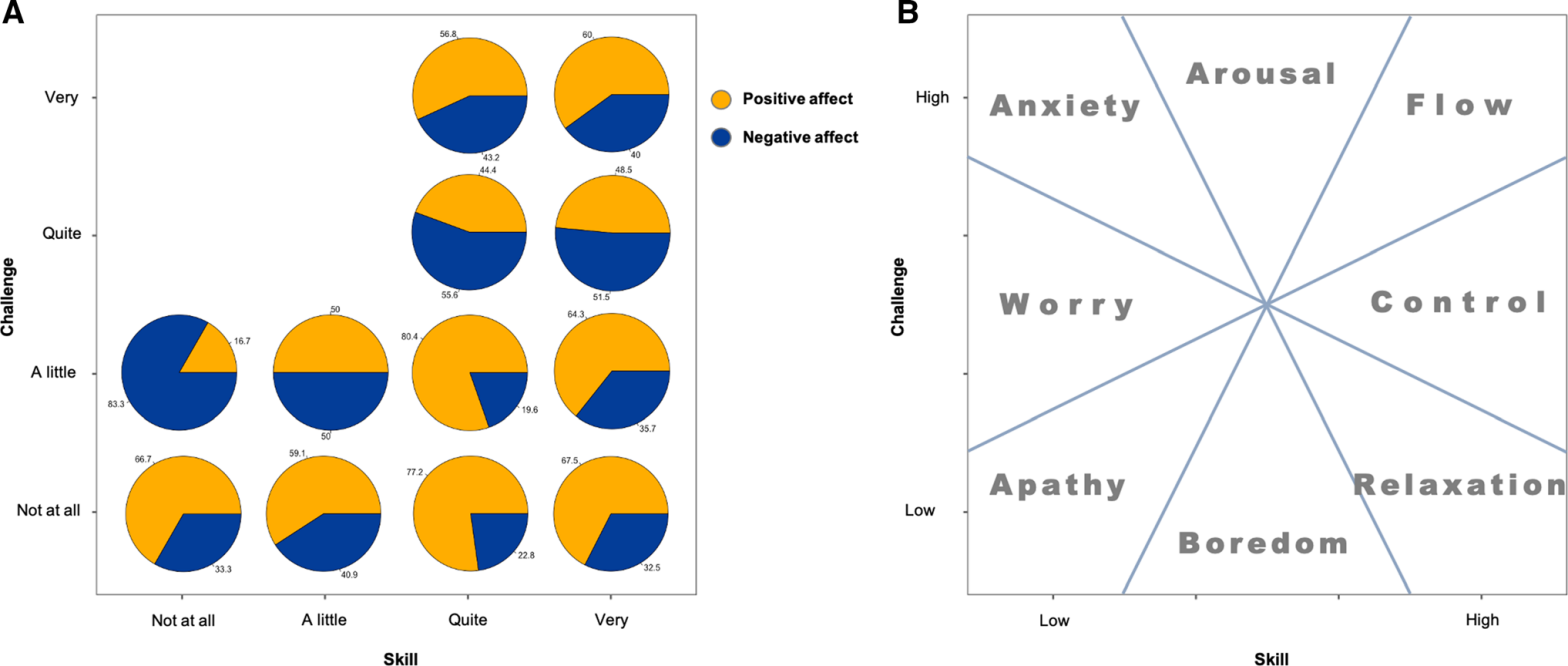
**A** Proportions of reported positive and negative affect according to the participant-assessed level of challenge and skill, excluding the combinations with *N* < 6. **B** Scheme of Csikszentmihalyi’s flow fluctuation model (Csikszentmihalyi and Csikszentmihalyi 1992) for reference

When combining participants’ answers to explore the occurrence of conditions for flow, we found that the most common combinations could be identified as either high challenge / high skill (*N* = 367) or low challenge/high skill (*N* = 334, Table S6). Activities reporting high challenge and high skill (theoretically related to flow state) were mostly in the category *fishing* (70%), *going fishing* (13%), and *returning from fishing* (11%) for very challenging, and *fishing* (42%), *going fishing* (11%), with various other activities accounting each for less than 10%, reported as quite challenging. *Non-fishing activities* (48%) and *resting* (28%) were the most commonly reported as not at all challenging. From those, 15% would be classified following the bivariate scheme of the flow model (Csikszentmihalyi and Csikszentmihalyi 1992) as apathy, while 85% corresponded to the state of relaxation/boredom. Relaxation/boredom was associated with the highest frequency of PA and lowest frequency of NA, and was consciously engaged in as shown by the main reason given, *I want to*. Combinations expected to lead to flow states, although mostly found in fishing activities, did not appear to be associated with more frequent PA than the other challenge/skill combinations, but on the contrary, PA and NA were almost equally frequent (Fig. 5).

## Discussion

Our results show that fishing activities were not directly associated with more frequent PA than non-fishing activities. Furthermore, although very low catch rates were associated with more frequent NA and less frequent PA, the highest levels of PA were reached at relatively low catch rates. While fishing activities were often associated with the combination of a high degree of challenge and perceived skill—the prerequisites of flow state—they in fact yielded less frequent positive affect and more frequent negative affect than non-fishing activities including rest.

The fact that fishing was not associated with more frequent PA than non-fishing activities, despite the identification of fishing as a source of well-being by fishers themselves, falsifies our first hypothesis. Our results show that positive affect was frequent during fishing preparation and decreased as the fishing trip progressed. Thus, the anticipation of going fishing, rather than fishing itself, was the only identifiable fishing-related activity that increased the frequency of fishermen’s PA. An especially negative affect-producing activity was selling the fish catch, which could be due to being exhausted after the fishing trip, stress about getting a low pay for their fish, or to low catches not being enough to fulfill their bond responsibilities (Miñarro et al. 2016; Sambuo et al. 2020). Besides, many fishermen complained that fishing takes place in very risky conditions and is dangerous due to monsoon storms, lack of safety equipment, and pirates, hazards that have also been reported elsewhere in Bangladesh (Kaplan and Kite-Powell 2000; Rahman and Schmidlin 2019). Thus, perhaps the expected idea of fishing is not matched by Bangladeshi fishermen’s reality, which echoes the discrepancy found in previous SWB studies between generic judgements of how enjoyable activities are, and actual episodic reports (Kahneman et al. 2004; Shiffman et al. 2008; Diener et al. 2009).

Second, while fishermen’s subjective assessment of a fishing trip was significantly related to their affect, the more objective assessment showed that the rate of fish catch was only related to affect over the lowest range of catch rates. The detailed emotions associated with fishers’ assessments of the fishing trip indicate that “worried” was the main emotion commonly reported when the fishing was not going well, and it was frequently associated with accidents regarding the boat engine, or nets getting tangled or lost. Such incidents may prevent the fishing trip from getting enough catch to cover the associated costs, which typically include payments to their patron or boat owner (Mozumder et al. 2019; Sunny et al. 2021), or may impair their ability to go fishing in subsequent days. But with the exception of such isolated accidents, we speculate that the stronger relationship between affect and fishermen’s own assessment of a successful fishing trip may reflect the importance of non-catch aspects, such as safety and good weather conditions, the absence of boat or gear malfunction, or good rapport with their crew mates. It is also possible that fishermen’s motivation for fishing influences their idea of “success”. In any case, our results empirically corroborate previous findings that—although catching something is important—fishermen do not need big catches to enrich their experienced well-being during or after fishing (Coulthard et al. 2011; Pollnac et al. 2012; Islam and Chuenpagdee 2018).

Third, the challenging and skillful activities, that might be associated with flow, were not associated with more frequent PA than the other states. Although caution must be made in directly associating the spatial array of our results with the more abstract model illustration, there is a clear trend toward more frequent PA in the lower right portion of Fig. 5A—positive affect dominates in situations that are not perceived as challenging. In contrast, negative affect is proportionally greater in more challenging situations, even at high self-assessed skill levels. It would appear that, for these fishermen, the most reliably positive situations occur when relaxing. This echoes findings from previous studies on momentary affect among western populations which found that leisure and relaxing activities topped the list of activities producing more frequent PA, as opposed to work, even for participants who found their job enjoyable and fulfilling (Kahneman et al. 2004; Shiffman et al. 2008). Consistent with this, most responses of ‘happy’ occurred at lower levels of challenge (Fig. S4). Furthermore, they consciously chose to engage in those activities, as shown by the predominant reason given, *I want to*. Conversely, the more challenging activities were undertaken only for the reason *I have to*, and although positive affective states were reported during these activities, they tended to be mild (‘well’, or ‘satisfied’) and were less common than worry (Fig. S4). Thus, although enjoyable flow states may have occurred for some fishermen during highly challenging fishing activities, they were not detectable by our method.

Our results suggest that previous findings that fishing provides overwhelmingly positive feelings of thrill and adventure in fishermen (Vittersø 1997; Pollnac and Poggie 2008) may either (a) not be significant among these Bangladeshi fishermen, or (b) be dimmed in the moment by other aspects. The previous studies were based largely on job satisfaction interviews, a method that differs substantially from ESM, and may be more sensitive to the constructed identity of fishers and other related values, rather than the momentary lived experience of the fishing activity. Flow theory further specifies that besides the balance between challenge and skills, activities should fulfill other criteria allowing the person to have a sense of control of the situation by understanding what needs to be done and how they are performing (Csikszentmihalyi 1990). Based on informal reports given by multiple inhabitants of the communities, we speculate that in Nijhum Dwip the dangers and economic pressures associated with fishing are too high, leading to a lack of control that make them often worried during fishing. In Chittagong, the more passive nature of the fishing strategy (i.e., leaving gillnets in a designated spot and doing several trips per day to and from the same fishing ground to pick up the catch and exchange the nets), may reduce the thrill of fishing and consequently the positive affect induced by flow states, supporting the idea that the reality of fishing in this fishery is far from an idealized concept of adventure and camaraderie.

Since fishing was viewed as one of the things that bring happiness in these communities (Miñarro et al. 2021), it could be assumed that fishermen value the fishing lifestyle. However, the status of commercial fishing has been declining in the last decades, with reported impacts on subjective well-being and job satisfaction of fishers across the world (Smith and Clay 2010). As societies develop and coastal communities transition from traditional fishing to more market-oriented livelihoods, some of the positive features of traditional fishing (community cohesion, camaraderie, and cultural ties) may be threatened by the demands stemming from a new focus on productivity and profit. In addition, top-down management from external actors, and having less control over the fishery, have been shown to lead to a powerlessness which contributes to the decline of subjective well-being in commercial fisheries (Smith and Clay 2010).

Finally, we caution that our method, despite relying on a well-established psychological tool, has several limitations that may bias our results. First, because of the intensive nature of the data collection, participants in ESM studies are likely to be more motivated, conscientious, agreeable and tech-savy than the general population (Diener 2009). For instance, our sampling design attracted mostly relatively young—and therefore less experienced—fishermen, and less years of fishing experience have been associated with a lower self-actualization from fishing and higher willingness to change their occupation (Pollnac et al. 2012). Second, our study considers only one activity of the many dimensions of small-scale fisheries that can potentially affect well-being of fishing communities. Many other activities have a paramount role in the well-being of other actors in small-scale fisheries, such as those derived by women through their role in dried fish value chains (Galappaththi et al. 2021), which deserve further consideration. Furthermore, the relational dimensions of well-being, including the fishers’ identity, camaraderie, and community cohesion are very relevant in small-scale fisheries and may account for certain discrepancies with other studies using more holistic measures such as life or job satisfaction. For instance, while many SWB studies have found differences in satisfaction with life between urban and rural settings (Easterlin et al. 2011), our sampling yielded no differences in affect between the sites. However, by examining the association between the fishing activity and affect in a focused manner, our work contributes to disentangle the mechanisms by which fisheries support the well-being of fishing communities.

## Conclusions

Despite the positive role of small-scale fisheries for community well-being, shown by previous works, our results question whether the fishing activity itself provides significant subjective well-being benefits, based on the studied small-scale fishermen in Bangladesh. Except for preparing to go fishing, fishing activities were not related to more frequent positive affect, even when meeting the conditions for flow (high challenge and high skill). On the contrary, several fishing activities were associated with more frequent negative affect than other daily activities, particularly when compared with resting and leisure. We also found that catching more fish was not a key factor in providing more frequent positive affect of fishers, although the self-assessed success of the fishing trip was associated with higher frequency of PA. Promising avenues for further research could explore other aspects of the quality of a fishing trip that may impact fishers’ subjective well-being. Fishing may not be particularly enjoyable in the moment, but it may provide a feeling of satisfaction, pride, and camaraderie that could raise life satisfaction, as well as contribute to the overall well-being of fishing communities. We speculate that government programs aiming to improve well-being in small-scale fishing communities may, therefore, be able to best achieve their goals by focusing on sources of positive identity and community dynamics, rather than trying to maximize fishing activities. Our results are encouraging in showing that effective management of small-scale fisheries, involving reductions in fishing effort essential to halt overfishing, would not be at odds with fishers’ subjective well-being, as long as other aspects about their livelihoods are improved.

## Supplementary Information

Below is the link to the electronic supplementary material.

Supplementary file1 (PDF 304 kb)
