# Supplementary material for: Does catching more fish increase the subjective well-being of fishers? Insights from Bangladesh
Source: Ambio. 2022 Feb 15;51(7):1673–86. doi: 10.1007/s13280-021-01698-5 (PMC9110605; doi:10.1007/s13280-021-01698-5)
Supplement: Supplementary file 1 — Supplementary file1 (PDF 304 kb) [file 13280_2021_1698_MOESM1_ESM.pdf]

Ambio

Supplementary Information

This supplementary information has not been peer reviewed.

Title: **Does catching more fish increase the subjective well-being of fishers? Insights from Bangladesh.**

Supplementary tables

Table S1. Summary of the ESM sample. Number of calls per site and age of participants.

Site	Data collection method	Sample size	Age
Nijhum Dwip	ESM by phone	590	25.1 ± 4.7
Chittagong	ESM by phone	485	31.7 ± 7.7

Table S2. Results from GLMM models for individual variables examined for effect on positive affect.

Variable examined	Chi-square	p
Activity	55.59	$9.4 \cdot 10^{-09}$ *
Site	0.001	0.972
Time of day	1.39	0.5
Call number	0.55	0.46
Physical discomfort	2.55	0.2795
Reason for doing activity	20.55	$3.5 \cdot 10^{-05}$ *
Self-assessed skill level of respondent	10.2	0.0372
Activity challenge degree	28.28	$3.2 \cdot 10^{-06}$ *
Subjective evaluation of fishing success	24.82	$4.1 \cdot 10^{-06}$ *
Fishing catch (kg of Hilsa)	1.38	0.24
Fishing efficiency (kg Hilsa per hour)	1.96	0.1613
Time fishing (hours)	0.34	0.56

Table S3. Results from GLMM models for individual variables examined for effect on negative affect.

Variable examined	Chi-square	p
Activity	49.35	$1.4 \cdot 10^{-07}$ *
Site	0.009	0.925
Time of day	0.16	0.9232
Call number	20.69	$5.4 \cdot 10^{-06}$ *
Physical discomfort	2.94	0.2302
Reason for doing activity	25	$3.7 \cdot 10^{-06}$ *
Self-assessed skill level of respondent	12.08	0.0168
Activity challenge degree	23.78	$2.8 \cdot 10^{-05}$ *
Subjective evaluation of fishing success	27.02	$1.4 \cdot 10^{-06}$ *
Fishing catch (kg of Hilsa)	0.0002	0.9878
Fishing efficiency (kg Hilsa per hour)	0.12	0.7253
Time fishing (hours)	0.01	0.91

Table S4. Results from GLMM models for individual variables examined for effect on physical needs.

Variable examined	Chi-square	p
Activity	28.78	0.0007 *
Site	24.62	$7 \cdot 10^{-07}$ *
Time of day	0.07	0.9644
Call number	34.77	$3.7 \cdot 10^{-09}$ *
Physical discomfort	61.85	$3.7 \cdot 10^{-14}$ *
Reason for doing activity	1.63	0.4421
Self-assessed skill level of respondent	38.26	$9.9 \cdot 10^{-08}$ *
Activity challenge degree	14.28	0.0026 *
Subjective evaluation of fishing success	1.31	0.5207
Fishing catch (kg of Hilsa)	0.77	0.3801
Fishing efficiency (kg Hilsa per hour)	7.29	0.0069 *
Time fishing (hours)	0.84	0.36

Table S5. Odds ratio table for the activities (with 95% confidence interval in parenthesis) for positive and negative affect. Asterisks denote statistically significant odds.

Activities	Positive affect	Negative affect
Fishing preparation	2.47 (0.65, 9.37)	0.62 (0.17, 2.36)
On the way to fish	0.72 (0.36, 1.45)	2.00 (0.99, 4.03)
Fishing	0.55 (0.34, 0.90) *	1.96 (1.19, 3.23)*
Net repair	0.75 (0.28, 2.00)	2.36 (0.89, 6.24)
Boat repair	0.19 (0.07, 0.50) *	3.51 (1.46, 8.47)*
Fishing-related unspecific	0.58 (0.18, 1.83)	1.42 (0.43, 4.66)
Return from fishing	0.51 (0.24, 1.10)	2.14 (0.99, 4.63)
Selling fish	0.33 (0.16, 0.67) *	3.81 (1.87, 7.78)*
Other activities	1.88 (1.11, 3.19) *	0.62 (0.36, 1.07)

Table S6. Proportions of positive affect, negative affect and main reason for doing activities for a selection of the main states implied by the flow model (Csikszentmihalyi and Csikszentmihalyi 1992).

Challenging	Skilled	Implied state	N	Positive (%)	Negative (%)	Reason
**Very**	Very	Flow	55	60	40	Have to (95%)
	Quite		136	55	43	Have to (86%)
**Quite**	Very	Flow	115	46	52	Have to (72%)
	Quite		61	55	44	Have to (59%), want to (33%)
**Very**	Not at all	Anxiety/worry	0	-	-	-
**Not at all**	Not at all	Apathy	22	62.5	37.5	Have to (55%), want to (32%)
**Not at all**	Very	Relaxation/ boredom	134	66	34	Want to (60%), have to (32%)
	Quite		200	79	21	Want to (60%), have to (26%)

Supplementary figures

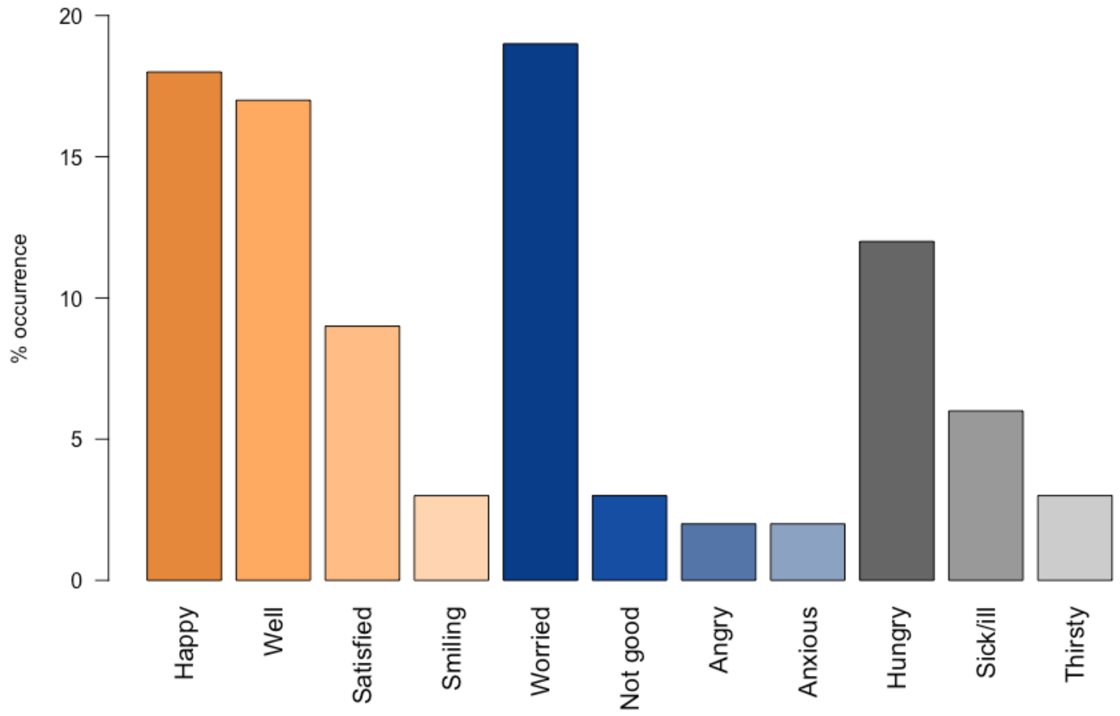

Figure S1. Proportions of individual emotions reported by fishermen.

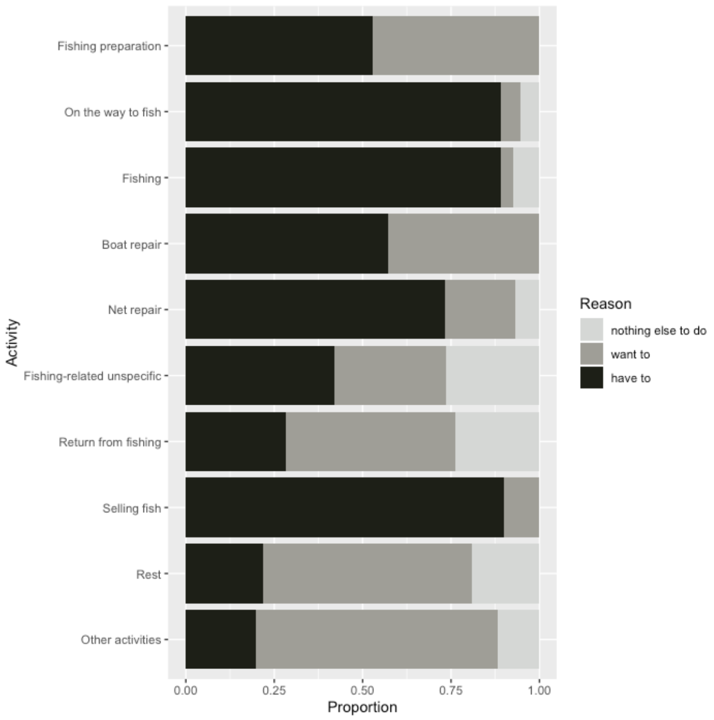

Figure S2. Reason for doing current activity reported by activity category.

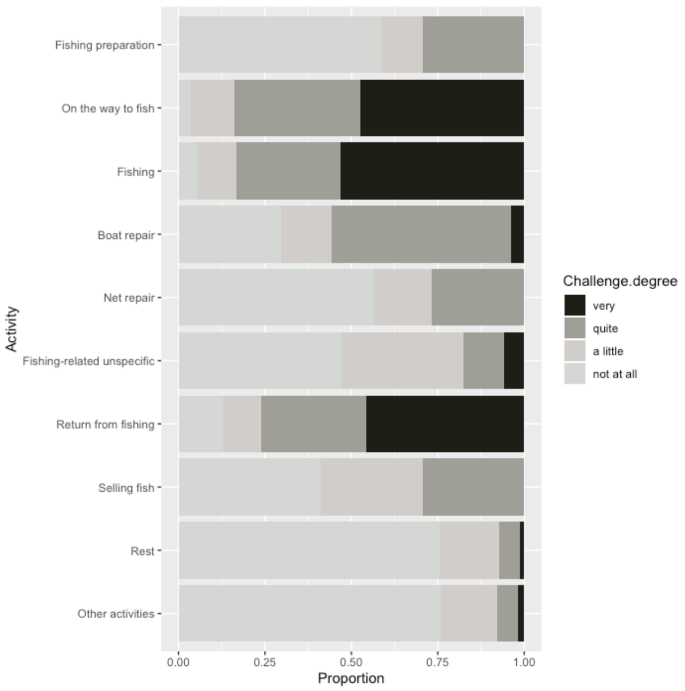

Figure S3. Activity challenge level reported by activity category.

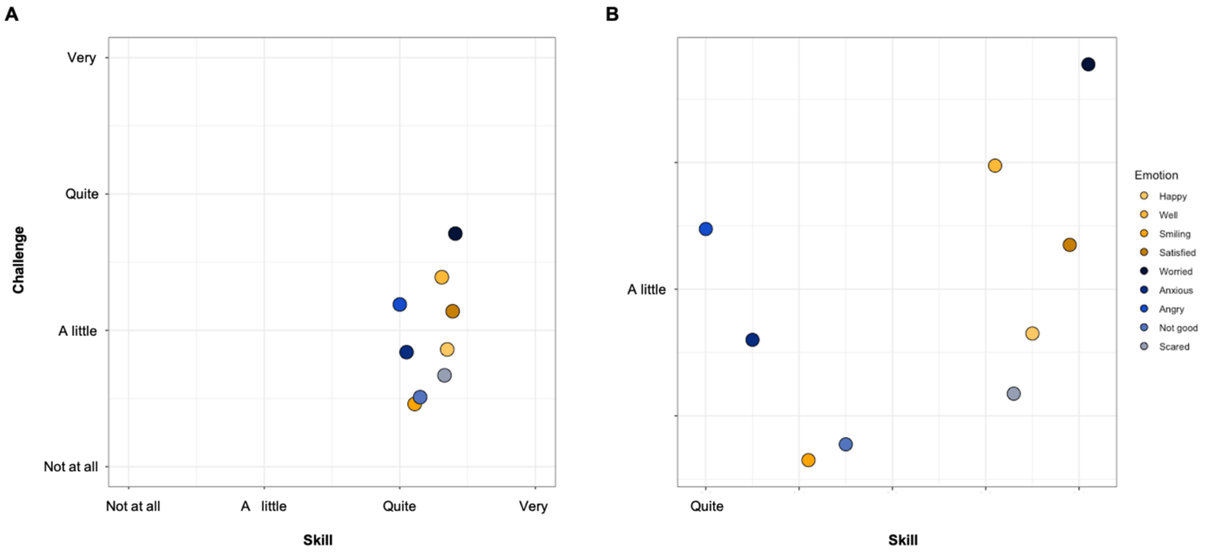

Figure S4. Reported individual positive and negative emotions mapped according to the mean challenge-skill balance associated to them by the studied Bangladeshi fishermen (A), and close up view of the emotions in their challenge-skill space (B).

ESM questionnaire

☐ Information summary
☐ Signed informed consent

Participant ID: ____________ **Date**: ___________________
Location: ________________ **Time**: ___________________
Interviewer: _______________

1- Where are you at this moment?

____

2- What are you doing right now?

____

3- Why are you doing this activity?

☐ I have to ☐ I want to ☐ I have nothing else to do

4- What are you feeling right now? Do you feel any of these emotions? (circle)

a. Satisfied
b. Happy
c. Smiling
d. Well
e. Scared
f. Worried
g. Not good at all
h. Angry
i. Ill, sick
j. Anxious
k. Hungry
l. Thirsty

5- Do you feel any physical discomfort at this moment?

☐ None ☐ A little bit ☐ Severe

How do you feel about this activity you are doing:

6- Is it challenging?

☐ Not at all ☐ A little ☐ Quite ☐ Very

7- Are you skilled at it?

☐ Not at all ☐ A little ☐ Quite ☐ Very

8- Do you wish you were doing something else?

☐ Not at all ☐ A little bit ☐ Very much

9- Who are you with?

☐ Alone ☐ Friends
☐ Male ☐ Female ☐ Mix
☐ Mother ☐ Father ☐ Siblings ☐ Children
☐ Strangers ☐ Other: ______________________

(how many? ____)

If fishing:

10-How long have you been fishing today: _______________minutes/hours

11-Did you catch anything? YES / NO

12-If yes, what have you caught (describe catch)?
____
____
____

13-Is the fishing good? Yes / No
